# Effects of Synthetic Fibers and Rubber Powder from ELTs on the Rheology of Mineral Filler–Bitumen Compositions

**DOI:** 10.3390/ma19010052

**Published:** 2025-12-23

**Authors:** Krzysztof Maciejewski, Witalij Zankowicz, Anna Chomicz-Kowalska, Przemysław Zaprzalski

**Affiliations:** 1 Faculty of Civil Engineering and Architecture, Kielce University of Technology, al. Tysiąclecia Państwa Polskiego 7, 25-314 Kielce, Poland; akowalska@tu.kielce.pl; 2 Recykl Organizacja Odzysku S.A., ul. Letnia 3, 63-100 Śrem, Poland; w.zankowicz@recykl.pl (W.Z.); p.zaprzalski@recykl.pl (P.Z.)

**Keywords:** end-of-life tires, rubber, fiber, pellet, asphalt mix, asphalt mastic, DSR, MSCR

## Abstract

**Highlights:**

**What are the main findings?**

- End-of-life tire (ELT) fibers and rubber powder enhance asphalt mastic rheology.
- Combined use of ELT fiber and rubber increases high-temperature stiffness and elasticity.
- ELT-derived additives improve recovery and reduce creep under load.
- Balanced fiber–rubber ratios maintain mid-temp flexibility of asphalt mastics.

**What is the implication of the main finding?**

- ELT-derived additives may improve sustainability of pavement materials.
- Improved durability and deformation resistance of asphalt mixtures.
- Possibility for reduction in need for polymer-modified binders and support for circular economy.

**Abstract:**

This study investigates the influence of synthetic fibers and rubber powder derived from end-of-life tires (ELTs) on the rheological behavior of asphalt mastics composed of paving-grade bitumen and mineral filler. Nine asphalt mastic formulations were prepared with varying fiber and rubber contents, reflecting the composition of stone mastic asphalt mixtures. Dynamic shear rheometer tests were conducted to assess dynamic stiffness modulus, phase angle, non-recoverable creep compliance, and elastic recovery. The results demonstrated that ELT-derived additives significantly enhanced high-temperature stiffness and elasticity, while maintaining satisfactory viscoelastic balance at lower temperatures. Synergistic effects between fibers and rubber were observed, improving both non-recoverable compliance and percent recovery, particularly at elevated shear stresses. Prolonged exposure to production temperatures (175 °C) confirmed the thermal stability of the modified mastics, with the most notable performance gains occurring during the first hour of heating. Based on the findings, it was concluded that ELT-based fiber–rubber additives can improve high-temperature performance of asphalt mastics without negative effects in intermediate and, possibly, also low service temperatures. This permits expanding the use cases for these kinds of additives beyond the role of inert stabilizers in stone mastic asphalt to an active modifier for extending asphalt mix performance.

## 1. Introduction

### 1.1. Asphalt Mixtures in the Framework of Circular Economy

With the continuous increase in traffic loads on road pavements, major efforts are being made to ensure the durability, quality, and sustainability of their construction and maintenance. Asphalt pavements make up most of the world’s road surfaces, and asphalt mixtures are highly suitable for reuse at the end of their service life. Currently, over 95% of asphalt mixtures in Europe are reused in some way, with up to 75% of them being used in new asphalt mixtures [1]. This creates strong potential for environmentally friendly asphalt technologies.

More than 80% of emissions associated with the entire life cycle of asphalt pavements are generated before the asphalt mixture leaves the production plant [2]—during the production and transport of raw materials and in the asphalt mixing process itself. These stages offer great optimization potential to enhance the ecological safety and sustainability of bitumen technologies, in line with the goals of the European Green Deal [3] and achieving carbon neutrality by 2050.

Promising directions for reducing emissions in asphalt pavement construction are possible due to new developments and increased understanding in the performance of cold mix [4,5,6,7], warm mix asphalt technologies [8,9,10,11,12,13], increasing the proportion of reclaimed asphalt in new mixtures, reducing moisture in raw materials (aggregates, sand, and RAP), and using bio-binders or other low-emission binders [14,15,16].

The impact of road construction industry on the environment can be reduced by increasing the durability and resilience of pavements, e.g., by utilizing polymer-modified asphalt binders, which exhibit superior fatigue properties and increased resistance to environmental factors [17,18]. In this scope, utilization of rubber and other components from recycled end-of-life tires (ELTs) or other recycled polymers can be seen as a feasible alternative to using virgin polymers. Such aim is highly reasonable, given the fact that over 40% of the ELTs (more than 1.4 million tons in Europe in 2019) are incinerated in cement kilns [19].

### 1.2. New Types of Additives Based on Fibers and Rubber from ELTs

In terms of ELT-based materials, currently, only crumb rubber and rubber powders are utilized in asphalt mixtures to some extent, mainly through the “dry” and “wet” mixing methods [20,21,22,23], but also new types of products such as preactivated rubber additives representing the “hybrid” asphalt mix modification are gaining traction [24,25].

The remaining component of the ELTs—textile and steel cords—remain mostly unused in this scope. Textile cord makes up about 5.5% of a passenger car tire’s weight, around 3% for light truck tires, and about 10% for SUV tires [26]. These cords are made from high-quality polyester, polyamide, aramid, and viscose fibers, which are strong and resistant to heat and chemicals. When surface-treated, they gain improved adhesion to rubber and other organic materials, including asphalt binders. Therefore, using this component of ELTs merely as an energy source is not the most efficient method of recycling.

A growing body of research highlights the benefits of utilizing textile cord fibers recovered from end-of-life (ELT) tires in asphalt mixtures, compared to their conventional disposal methods such as combustion in cement kilns or landfilling [26,27,28]. According to [28], incorporating synthetic fibers from ELTs into porous asphalt and stone mastic asphalt (SMA) mixtures can enhance fatigue resistance by up to 70% while maintaining the technological performance verified through binder drainage tests. When compared to cellulose-fiber-stabilized mixtures, this approach can also reduce the overall environmental impact by as much as 15%, based on assessments using Cumulative Energy Demand (CED), Global Warming Potential (GWP), and the ReCiPe model.

In gap-graded mixtures, textile fibers derived from ELTs can function similarly to cellulose-based additives by preventing material segregation [29,30] and simultaneously improving water resistance [30]. Owing to their lower binder absorption capacity, these fibers also enable a reduction in binder content without compromising the mechanical performance of the asphalt mixture [30].

Furthermore, incorporating textile fibers from ELTs has been shown to enhance crack resistance [31,32], primarily by slowing the progression of microcracks into macrocracks [33]. However, in mixtures with a thinner asphalt film, careful optimization of the binder content is necessary to ensure adequate water resistance [18]. Studies involving combinations of textile fibers from ELTs and rubber powder mixed with bituminous emulsion have demonstrated notable improvements in the fatigue life of both asphalt concrete (AC) and SMA mixtures [34].

Research on the rheological behavior of asphalt binders containing textile fibers from used tires indicates enhancements in the rutting parameter (G*/sinδ) and improved recovery characteristics in multiple stress creep recovery (MSCR) tests [35]. These findings were corroborated by asphalt mix testing in high service temperatures [32].

The use of tire-derived materials—particularly granulated rubber combined with synthetic textile fibers from ELT cords—in the production of asphalt mixtures was also previously investigated in Poland [36,37].

Despite promising results, ELT textile cord-based additives are not widely used due to difficulties in fiber cleaning, processing, and uniform dosing. Fibers obtained directly from tire recycling are typically heterogeneous, loose, and braided, with highly variable properties. Producing an additive with stable, well-controlled properties and a form that enables consistent dosing requires careful selection of raw materials and the use of a specialized processes [38].

A practical solution is the recently developed SMAPOL^®^ additive (prod. Recykl O.O. S.A.), which functions as a stabilizing and reinforcing component in mineral–asphalt mixtures. Its proprietary production process enables strict control over the additive composition, obtaining robust pellets, and optional incorporation of adhesion promoters, rejuvenators, waxes, and other modifiers. The performance of such additives can be tailored through fiber length, rubber powder content, and particle size, and optional impregnation with auxiliary agents. However, current research offers limited guidance on optimizing the composition of such composite additives, largely due to challenges in controlling their variable properties.

### 1.3. Scope and Significance of the Study

This study evaluates the rheological properties of asphalt mastics composed of paving-grade, unmodified binders with mineral filler, supplemented with textile fibers from ELTs and fine rubber powder. Recently, a proprietary method was developed, enabling us to form the evaluated fibrous and rubber components based on ELTs into robust pellets, permitting widespread adoption of this type of additive in asphalt mix production. At the moment, this method is used for producing an anti-drainage (binder drainage) additive for stone mastic asphalt; however, the results in mixture testing indicate possibilities for significant improvements in mix performance if the additive is optimized.

Asphalt mastic was selected instead of neat binder because relatively large additive particles strongly affect its physical behavior and, consequently, mixture performance [39,40,41]. Interest in asphalt mastics is also increasing due to their relevance in understanding the mechanical behavior of asphalt mixtures [42]. The investigated mastics were designed to closely represent the composition of stone mastic asphalt (SMA).

The experimental mastics were produced without specialized homogenization, following a process analogous to the industrial “dry” modification method. This preserved the reinforcing function of the fibers by avoiding size reduction or dissolution, with only deagglomeration performed to ensure uniform distribution. Limestone filler, rubber powder, and synthetic fibers were added simultaneously to the binder.

The results of this research are expected to provide a basis for developing effective methodologies for optimizing the composition and manufacturing of high-quality granular fibrous additives, applicable to a wide range of asphalt mixtures intended for various purposes.

## 2. Materials and Methods

### 2.1. Materials

The composition of the investigated asphalt mastics reflected the relationships between the mineral filler and asphalt binder in a SMA 11 wearing course stone mastic asphalt mixture designed in line with the national guidelines [43]. Based on the material proportions in the referenced SMA mixture, a 1:1.79 ratio of asphalt binder to the calcareous mineral filer was used. The asphalt binder utilized in this study was a paving-grade bitumen of a 50/70 type (penetration at 25 °C: 64.1 ∙ 0.1 mm; softening point: 47.8 °C).

The base materials for obtaining the ELT-derived additives used in the study were obtained by mechanical grinding of passenger car tires at ambient temperatures. The two investigated materials included synthetic fibers (F) and rubber powder (R). The ELT-derived additives used in the study were produced on an industrial scale using a proprietary process, its outline provided below.

**Figure 1:**
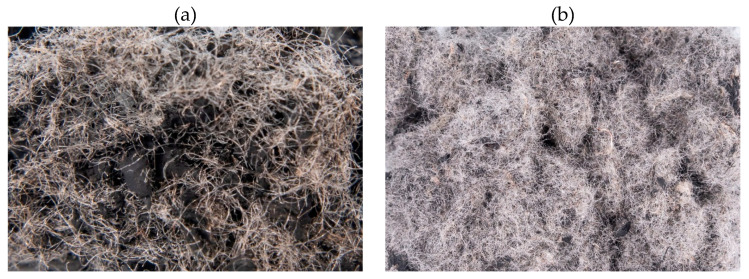

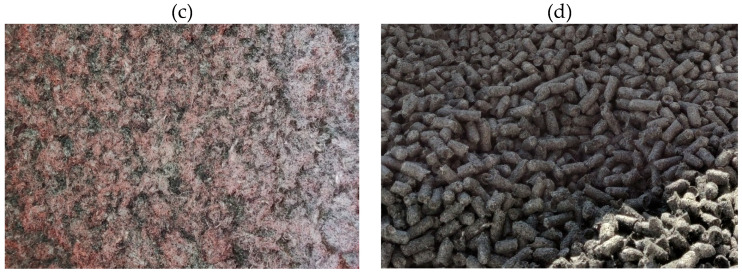
ELT textile cord after consecutive processing steps: (**a**) raw textile cord (avg. length 13 mm); (**b**) textile cord after cleaning; (**c**) agglomerated and activated textile cord; (**d**) pelletized textile cord (length 25 mm, diameter 6 mm).

The fibrous material on different stages of processing is shown in Figure 1. These steps included obtaining the raw textile cord from the crumb rubber production derived from end-of-life tire recycling (Figure 1a), removal of impurities (Figure 1b), agglomeration of the fibers with their activation (Figure 1c), and pelletization (Figure 1d). Due to the specifics of the processes used, the quality of textile cord material can be accurately controlled, and the fibrous material is formed into uniform pellets without using any additional binding agents, enabling easy and convenient use of this material in the plant scale production of asphalt mixtures.

The rubber powder used in this study was derived from the purification process of the tire cord material described above. The rubber powder was agglomerated, partially activated, and granulated.

The basic properties of the ELT-derived additives are provided in Table 1. Although both components (fiber and powder) undergo cleaning process, some residual rubber powder is present in the pelletized fibers and some fiber content is contained in the rubber powder. Additionally, the pelletized fibrous material can be supplemented with additional rubber powder and/or other components if needed.

### 2.2. Sample Preparation

The evaluated ELT-derived additives can be considered to be active, and their properties are expected to change with the action of high-temperature mixing and due to interactions with the asphalt binder. For this reason, the experimental design and sample preparation protocol were developed to realistically reproduce the production conditions of stone mastic asphalt mixtures and the conditions applied in the binder drainage test, in accordance with EN 12697-18 [44] standard.

First, the ELT-derived additives were deagglomerated using a high-speed blade shredder (Bosch TSM6A013, Robert Bosch, Gerlingen, Germany, rotational speed: 17,000 rpm, blunt blade). Each batch consisted of 15.0 ± 0.5 g of material, processed for 10 s. The procedure was repeated until the required quantity of loose, non-agglomerated fibers and rubber particles was obtained.

The asphalt binder and mineral filler were preheated in a ventilated oven to a temperature that ensured a target final mixing temperature of 180 ± 5 °C after the addition of room-temperature fibers and rubber powder.

Mixing was performed manually under low-shear conditions using a glass rod to avoid mechanical degradation of the fibers. First, the binder was combined with the preheated mineral filler and vigorously mixed for 15 ± 2 s until a homogeneous mastic was obtained. Subsequently, the pre-deagglomerated ELT-derived additives were added and mixed for an additional 30 ± 10 s until visible uniform distribution of fibers and rubber particles was achieved.

When required by the experimental design, freshly prepared mastics were transferred immediately to a ventilated oven and conditioned at 175 °C for 1 h or 2 h. During heat conditioning, samples were manually stirred every 30 min for approximately 10 s to limit particle sedimentation and ensure thermal homogeneity.

**Figure 2:**
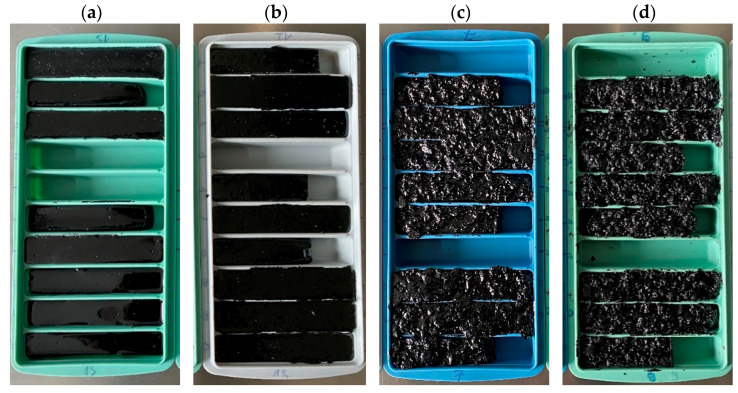
Selected asphalt mastics containing different amounts of ELT-derived additives (F: synthetic fiber, R: rubber powder) after mixing: (**a**) F:0%, R:0%, (**b**) F:0%, R:4%, (**c**) F:8%, R:0%, (**d**) F:8%, R:4%.

After conditioning, the mastics were poured into silicone rubber molds (Figure 2) and allowed to cool at ambient room temperature for a minimum of 24 h. Test specimens for dynamic shear rheometer (DSR) measurements were prepared by trimming and partitioning the cooled material using a sharp-edged cutting tool, without reheating, in order to avoid additional thermal history effects.

A total of nine asphalt mastic compositions were investigated. The compositions differed in the content of synthetic fibers (F) and rubber powder (R), ranging from a reference mastic without additives to a maximum total additive content of 16% by mass of binder. Four compositions were tested in three different thermal states: immediately after mixing, after 1 h of conditioning, and after 2 h of conditioning at 175 °C. The remaining compositions were tested after 1 h of thermal conditioning.

### 2.3. Methods

The rheological properties of asphalt mastics were evaluated using a dynamic shear rheometer. The evaluated parameters included the following:

− Dynamic shear modulus (|G*|) and phase angle (δ) determined in accordance with EN 14770 [45], at temperatures from 10 to 90 °C in steps of 10 °C, at frequencies from 0.16 to 16 Hz under controlled strain;
− Multiple stress creep recovery tests carried out in accordance with the EN 16659 [46] at a temperature of 60 °C, in sequences of increasing shear stress values of 0.1, 1.6, 3.2, 6.4, 12.8, and 25.6 kPa.

The presented results of oscillatory DSR tests represent averages obtained from two samples in the respective tests. Additional samples were tested when the limits for repeatability provided by the testing standards were exceeded. In the MSCR tests, four samples were tested and 95% confidence intervals were calculated (represented by the error bars in the figures).

## 3. Results

### 3.1. Temperature Stability of the Asphalt Mastics with ELT-Derived Additives

The temperature stability of the investigated asphalt mastics was evaluated to check for possible deterioration of their rheological properties after prolonged exposure to high temperatures (representative of mix production, transportation, and paving). In this part of the study, four selected asphalt mastics were subjected to oscillatory and MSCR tests after different heating times:

− 0 h, directly after mixing of the components of the mastics;
− 1 h, corresponding to the transport of asphalt mix;
− 2 h.

**Figure 3:**
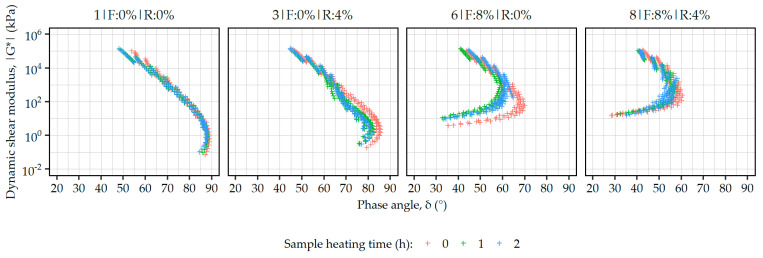
Black diagrams representing the changes in the rheological properties of the selected asphalt mastics due to their prolonged exposure to high temperatures.

Figure 3 presents the evolution of rheological characteristics of the mastics #1, #3, #6, and #8 in the form of Black diagrams (|G*| vs. δ) after the above-mentioned time intervals of exposure to elevated temperatures.

In the case of the formulation #1, consisting only of bituminous binder and mineral filler, its prolonged heating resulted only in limited changes to the relationships between the dynamic shear modulus and the phase angle. Small shifts in the curve due to the increases in the |G*| values and lowering of the phase angles could be observed at the top and bottom of its ends of the curve (|G*| values in ranges of ca. 10^2^–10^5^ and 10^−1^–10^0^ kPa, respectively). The probable causes of these changes include absorption and adsorption of the binder by the filler particles and its oxidative aging. It should be noted that the curves obtained after 1 and 2 h of heating of the samples differed minimally.

Formulation #3 directly after mixing (0 h) presented a similar characteristic to the previously discussed plain mastic, with only a small decrease in phase angles and small overall increase in stiffness, which could be attributed to the presence of additional particles of rubber powder and decreased proportion of bituminous binder in the mastic. After heating for 1 h though, a significant shift in the curve shown in the Black diagram is observed in the intermediate to low |G*| region (below 10^3^ kPa), mainly due to decreases in the phase angle values. This effect can be explained by the activation of the ELT-derived additives present in this formulation, and the resultant shape of the Black curve is similar to those observed in some polymer-modified bitumens [47,48]. Further heating of the material for a total of 2 h did not result in any additional significant changes in the evaluated characteristic.

The formulation containing 8% of the synthetic fiber material (#6) exhibited drastically different rheological behavior, as shown in the Black diagram, with significantly reduced phase angles and similarly increased |G*| values at high temperatures. In this case, the exposure to high processing temperatures resulted in decreases in phase angles, again, most prominent below 10^3^ kPa dynamic shear moduli values. The Black curves obtained after 1 and 2 h of exposure to high temperatures were similar, with slightly decreased phase angles after 2 h.

Similar characteristics were recorded in the case of formulation #8, which additionally contained 4% of the rubber powder. Here, the Black curves were further shifted towards lower phase angle values, with slightly higher values of the dynamic shear moduli. Compared to formulation #6, this material exhibited smaller changes when subjected to high temperatures in the 1 and 2 h intervals, and also the most visible effects included the decrease in the phase angles.

The selected oscillatory results presented in Table 2 confirm the observations made based on the Black diagrams. The asphalt mastics subjected to prolonged exposure to high temperatures underwent consistent changes in terms of dynamic shear modulus and phase angle across the testing temperatures, with increases in |G*| and decreases in δ observed with increased heating time, with only a small deviation seen in the case of formulation #6 after 2 h of heating.

A similar picture of the temperature stability can be drawn from the MSCR data, also presented in Table 2. In almost all cases, the non-recoverable creep compliance decreased significantly when the mastics were subjected to 1 h of heating, with a further (but smaller) decrease observed after total 2 h heating time. Only in the case of plain asphalt mastic (#1), a small increase in J_nr_ values was observed at the 2 h mark.

The obtained recovery values of the plain asphalt mastic (#1), regardless of its heating time, can be safely categorized as too small to be considered significant in engineering terms. On the other hand, the mastics containing the ELT-derived additives exhibited significant elastic response even directly after mixing (0 h), which increased as the result of prolonged heating.

### 3.2. Effects of the Asphalt Mastics’ Composition with ELT-Derived Additives on Their Rheological Properties

#### 3.2.1. Performance in Oscillatory Tests

Based on the oscillatory test results in which the dynamic shear moduli were determined, complex stiffness modulus mastercurves for each of the tested asphalt mastic formulations were constructed by shifting the data measured in respective temperatures to new frequencies (f_r_) at a reference temperature (T_ref_ = 50 °C) set in the midpoint of the obtained data. The mastercurves were produced using a sigmoidal model (Equation (1)). The relationship between the actual and reduced frequencies is given by Equation (2), while the shift factors α_T_ were defined through the Williams–Landel–Ferry relationship given by Equation (3).

(1) $$\log\left(\left|G^{*}\right|\right)=\nu +\frac{\alpha }{1+e^{\beta +\gamma logf_{r}}}$$

(2) $$log\;f_{r}-log\;f=log\;a_{T}$$

(3) $$\log\left(\alpha_{T}\right)=\frac{C_{1}(T-T_{0})}{C_{2}+(T-T_{0})}$$

The shifting of the experimental data was conducted by using a nonlinear least-squares method [49] to obtain best fit of the sigmoidal model (Equation (1)).

**Figure 4:**
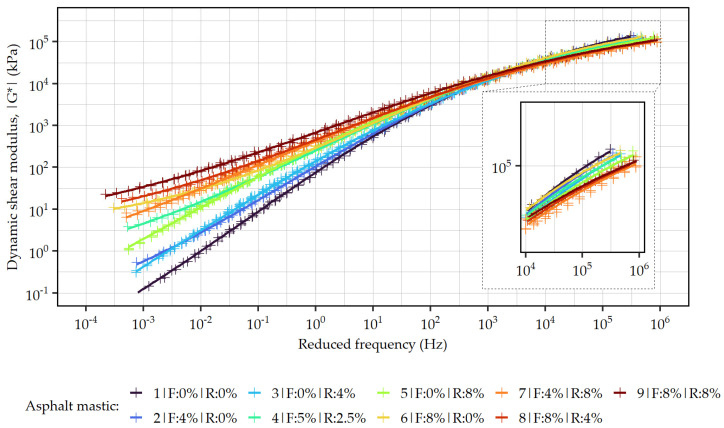
Dynamic shear modulus mastercurves of asphalt mastics with different amounts of ELT-derived additives.

Table 3 presents the fitting parameters of the dynamic shear modulus mastercurves for all the tested asphalt mastics, computed at a reference temperature of 50 °C (midpoint of the testing range), while their graphical representation is provided in Figure 4. In all cases, a very good fit of the experimental data to the sigmoidal function (Equation (1)) was achieved, as shown by high values of the coefficient of determination (R^2^ > 0.98).

The increasing content of the ELT-derived additives significantly impacted the low-frequency (high-temperature) portion of the mastercurves, increasing the |G*| values in this portion of the curves. The curves obtained with formulations #3, #5, #7 were shifted upwards at low frequencies, but remained approximately parallel to the mastercurve of the plain asphalt mastic (#1)—these formulations contained either only the addition of rubber powder, or, as in the case of #7, its content was higher than that of the synthetic fibers. In the case of the remaining formulations (#2#, #4, #6, #8, #9), with dominating fiber content), the mastercurves in the low-frequency region were not only shifted towards higher |G*| but were also curved upwards, indicating significant effects to the low-temperature asymptote. At high loading frequencies, the observed variation in the mastercurves was significantly smaller. Higher contents of the ELT-derived materials yielded lower values of dynamic shear moduli and so did the higher contents of the synthetic fiber component of the additive. These effects can be verified by the values of the lower and upper modulus asymptotes calculated based on the fitted ν and α parameters of the mastercurves (Table 3). A particularly strong effect on the position of the lower mastercurve asymptotes was registered in formulations #6, #8, and #9 containing the highest amount of the synthetic fiber component, yielding an increase measured in multiple orders of magnitude.

**Figure 5:**
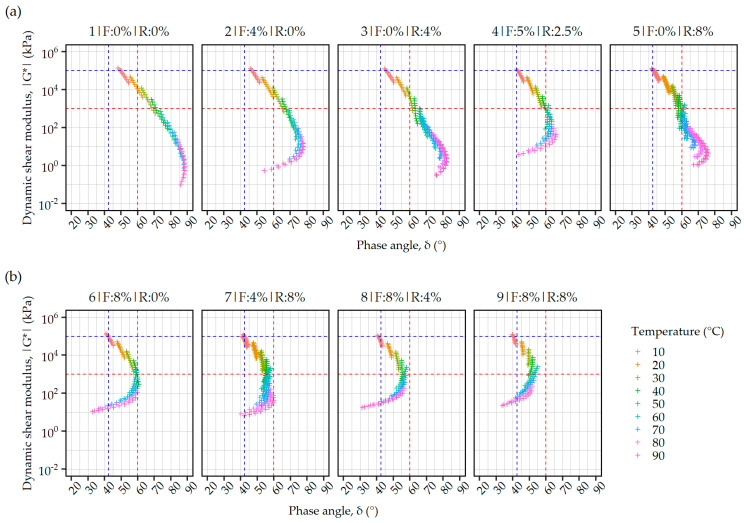
Black diagrams representing the effects in dynamic responses of the selected asphalt mastics containing different amounts of ELT-derived additives: (**a**) formulations #1–#5, (**b**) formulations #6–#9.

Further observation can be made based on the analysis of the Black diagrams of the asphalt mastic formulations shown in Figure 5. Here, not only changes in the dynamic shear moduli, but also in the values of phase angle depending on the contents of the ELT-derived additives and the testing temperature, can be evaluated. Similarly to the mastercurve analysis, the most prominent variation in the rheological character of the mastics was observed in intermediate and high temperatures, typically above 40 °C (|G*| below 10^3^ kPa). At those higher temperatures, major changes in both the dynamic shear moduli and phase angles can be observed with the increasing amounts of the additives. In most cases, consistent increase in the |G*| and decrease in the phase angle values were recorded when the synthetic fiber and rubber powder content increased. Only in the case of formulations #3 and #5 (with sole addition of rubber powder) were higher values of the phase angles and lower dynamic stiffness moduli observed.

Based on the shape of the Black curves in the low-temperature region, three distinct groups of the asphalt mastics can be distinguished. The first group (comprising formulations #1–#3) constitutes mastics with low-temperature performance, which was weakly influenced by the additives. The second group (formulations #4–#8) was characterized by decreased phase angles and nearly identical characteristics in the low-temperature region contained in the space marked in the figure with the red and blue dashed reference lines. The third group comprising formulation #9 was characterized by phase angles even more strongly decreased in this region. It should be noted that excessive decrease in phase angle at low temperatures could result in increased brittleness of the investigated material due to the loss of the viscous component in the stiffness modulus. Based on these observations, it can be stated that the increase in the content of ELT-derived materials in the formulations #4–#8 had limited effects on the low-temperature performance of the asphalt mastics (as seen in the region marked by the reference lines), while the high-temperature performance was continuously improved.

#### 3.2.2. Creep Stress Sensitivity Evaluated in Multiple Stress Creep Recovery Testing

**Figure 6:**
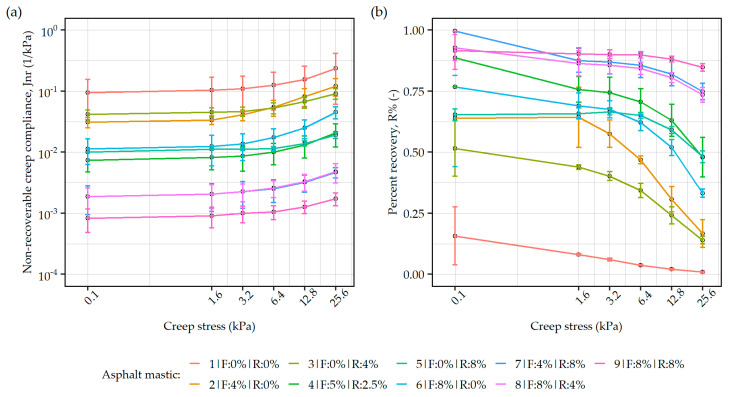
Results of multiple stress creep recovery testing of asphalt mastics with different amounts of ELT-derived additives subjected to different values of creep stress: (**a**) non-recoverable creep compliance, (**b**) percent recovery.

Figure 6 shows the creep recovery behavior of the asphalt mastics recorded in MSCR testing under increasing shear stress levels. Based on the non-recoverable creep compliance values (Figure 6a), the results of the mastics containing ELT-derived materials can be roughly clustered into four groups (omitting the plain asphalt mastic #1) containing increasing amounts of the additives: (1): #2 and 3#, (2): #4, #5 and #6, (3): #7, and #8, (4): #9. Group (1) with 4% of either synthetic fiber or rubber powder was characterized by the highest values of J_nr_, second only to the plain asphalt mastic. The second group, consisting of formulations with intermediate amounts of the ELT-derived material amounting to 7.5–8% also exhibited intermediate values of the non-recoverable creep compliance. The two asphalt mastics in group (3) with additive contents adding up to 12% performed in a very similar manner, regardless of the type of the dominating component (synthetic fiber, rubber powder). The lowest J_nr_ values could be attributed to formulation #9, with the highest concentration of the synthetic fiber and rubber powder. The consecutive groups of asphalt mastics containing the ELT-derived additives differed in the J_nr_ values significantly (approx. 10× per group), possibly indicating an excessive increase in the viscosity of the tested material, whose effect should be verified at production temperatures.

Based on the analysis of the results in percent recovery (R%) presented in Figure 6b, the investigated mastics with ELT-derived additives were discriminated into three groups: (1): #2, #3 and #6, (2): #4 and #5, (3): #7, #8 and #9. Group (1) exhibited intermediate values of percent recovery up to 3.2 kPa creep stress (30% < R% < 75%), but with increased stress levels, the R% fell below 30% at the end of testing. Group (2) was characterized by a similar magnitude of percent recovery at low creep stresses but retained high values of this characteristic (R% > 50%), even under maximum shear loading. The best-performing group (3) showed very high recovery values (R% > 75%) under all evaluated stress levels. This clustering is similar to that conceived based on the J_nr_ values, with the main change in the grouping of formulation #6. This shows that the elastic recovery potential of the mastics may be more significantly more affected by the rubber powder component than the fibrous one.

**Figure 7:**
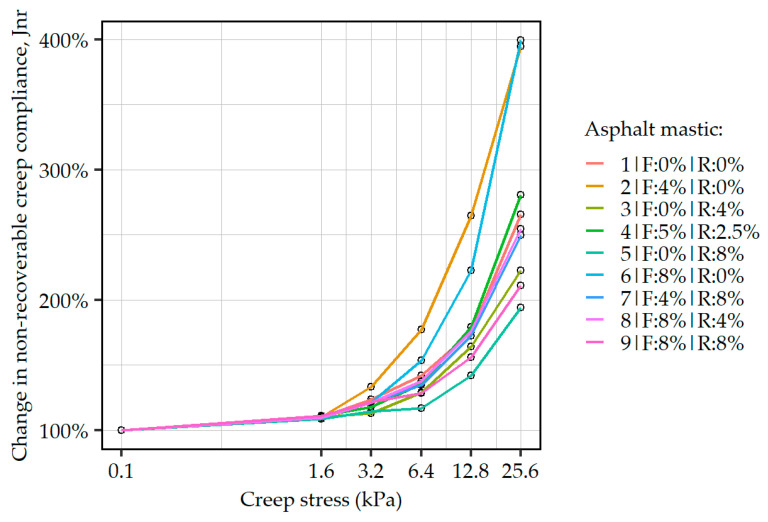
Effects of increasing creep stress on the non-recoverable creep compliance of the asphalt mastics with different amounts of ELT-derived additives.

Figure 7 presents the non-recoverable creep compliances measured at different creep stress in relation to the J_nr_ (0.1 kPa) values (measured at 0.1 kPa creep stress), allowing us to comparatively evaluate the effects of increasing creep stress on this characteristic, revealing potential susceptibility of the asphalt mastics to more demanding loading conditions. Here it was found that the formulations containing only the synthetic fiber additive exhibited increased susceptibility to creep stresses exceeding 3.2 kPa, while the smallest changes in J_nr_ values under increasing shear loading were related to the mastics in which the rubber powder component dominated or was very high (#5, #9, #3). In view of these results, it can be stated that for a given level of non-recoverable compliance, the fibrous component introduces a potentiality for decreased creep performance in high shear scenarios, while the rubber powder component inhibits it. It can be postulated that one of the reasons for this behavior lies in the shape of the main constituents of these components—the synthetic fibers with their length as the major dimension may introduce slip surfaces in the mastics, while the rubber powder with irregular spherical shapes counteracts this phenomenon.

#### 3.2.3. Statistical Modeling of Oscillatory and MSCR Characteristics of Asphalt Mastics with ELT-Derived Additives

The effects of the composition of the ELT-derived additives on the properties of the asphalt mastics were evaluated by applying two-variable, first-order (linear terms) and second-order (quadratic terms) statistical models with interaction of the linear terms shown in Equation (4), where y: predicted response, $\beta_{0}$: intercept, $\beta_{i}$: linear coefficients (F, R), $\beta_{ii}$: quadratic coefficients (F^2^, R^2^), $\beta_{ij}$: interaction coefficient (F:R), ε: random error term. First-order and second-order models were applied adequately to reveal the statistical significance of the independent variables.

(4) $$y=\beta_{0}+\beta_{1}x_{1}+\beta_{2}x_{2}+\beta_{11}x_{1}^{2}+\beta_{22}x_{2}^{2}+\beta_{12}x_{1}x_{2}+\varepsilon$$

Table 4 and Table 5 present the fitted parameter values and the evaluation of their statistical significance for predicting log_10_(|G*|) and phase angle measured at temperatures from 10 to 90 °C at frequency of 1.59 Hz, obtained by the means of ANOVA procedure. For the purpose of statistical modeling, the |G*| values were subjected to logarithmic transformation due to the exponential nature of this parameter.

Based on the results of the statistical analysis of the data, it can be observed that the contents of the ELT-derived additives did not significantly influence the values of the dynamic shear moduli up to the temperature of 20 °C. At temperatures higher than 20 °C, both the synthetic fiber and rubber powder significantly affected this parameter. Additionally, at temperatures higher than 40 °C the interaction between these components was also found to be statistically significant. Conversely, in the case of the phase angle, both variables (synthetic fiber and rubber powder content) as well as their interaction were statistically significant up to the temperature of 70 °C. At higher temperatures, the phase angles of the investigated mastics were more strongly affected by the content of synthetic fiber. These observations are in line with the analysis conducted based on the dynamic modulus mastercurves and Black curves.

Table 6 and Table 7 present the fitted parameter values and the evaluation of their statistical significance for predicting log_10_(J_nr_) and R% obtained at 60 °C by the means of ANOVA procedure at different creep stresses. For the purpose of statistical modeling, the J_nr_ values were subjected to log transformation due to the exponential nature of this parameter, alike the dynamic modulus values. Both the synthetic fiber and rubber powder content were statistically significant (*p* ≤ 0.05) in terms of shaping the values of the independent variables at all levels of creep stress. In the case of the non-recoverable creep compliance, the statistical significance of the interaction term was confirmed only at the highest creep stress. Conversely, in the case of the percent recovery R%, this was the only case when the interaction term was not found to be statistically significant.

**Figure 8:**
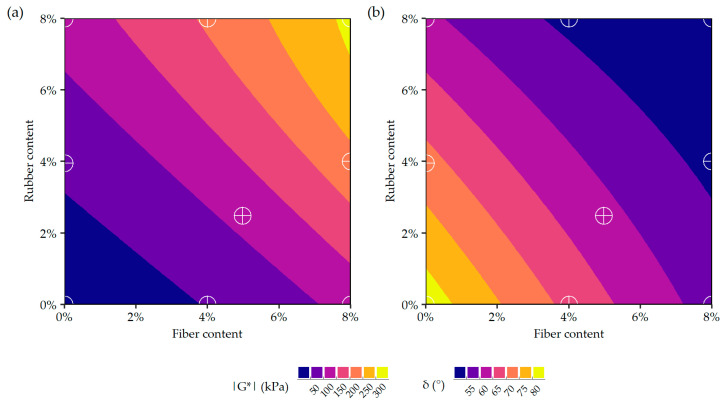
Graphical representations of the statistical models for predicting the oscillatory characteristics of asphalt mastics with ELT-derived additives at 60 °C and frequency of 1.59 Hz: (**a**) dynamic shear modulus (|G*|), (**b**) phase angle (δ).

**Figure 9:**
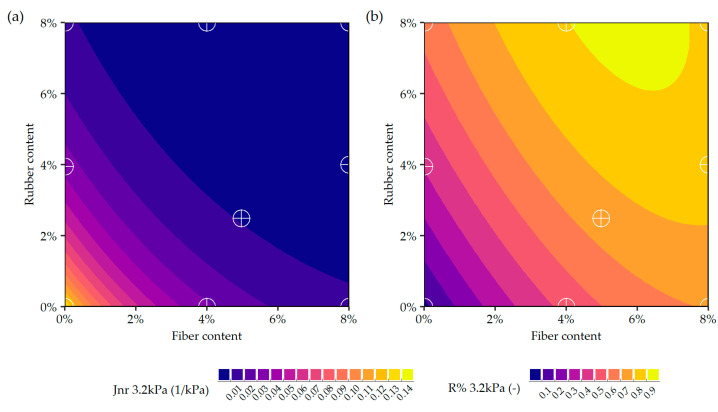
Graphical representations of the statistical models for predicting the MSCR characteristics of asphalt mastics with ELT-derived additives at 60 °C and 3.2 kPa of creep stress: (**a**) non-recoverable creep compliance (J_nr_), (**b**) percent recovery (R%).

Graphical representation of the selected statistical models predicting the rheological characteristics of the asphalt mastics are presented in Figure 8 and Figure 9.

## 4. Discussion

The presented results demonstrate that incorporating ELT-derived additives—synthetic fibers and rubber powder—significantly influenced the rheological performance of the investigated asphalt mastics based on a 50/70 paving-grade bitumen. The compositions of the investigated material resembled asphalt mastic of a wearing course SMA mixture. Although, typically, fiber-based additives in SMA mixtures are only used to prevent binder drainage and to assure mix homogeneity, it was found that ELT-derived synthetic fibers and rubber powder can improve functional properties of asphalt mastics and, presumably, asphalt mixtures as a result.

The obtained dynamic shear modulus mastercurves and Black diagrams confirmed that the simultaneous use of both ELT-derived additives permits increasing the high-temperature stiffness of the binder/mastic system, while maintaining a stable level of viscoelastic balance at lower temperatures. The synthetic fiber component enhanced the elastic response of the mastic, as evidenced by the observed decrease in phase angle and increase in complex modulus in the intermediate-to-high temperature range. In MSCR tests, the high fiber content provided not only improvement to the non-recoverable compliance but also introduced significant recovery values. It was, however, observed that the mastic formulations based only on the synthetic fiber component were visibly more susceptible to creep under higher shear stresses. The rubber powder used on its own also improved overall elasticity and temperature stability of the asphalt mastics—the Black curves of formulations including only addition of the rubber powder resembled polymer-like modification and, at the same time, the multiple stress creep recovery tests showed improvements in both evaluated parameters (J_nr_ and R%). Simultaneous use of both ELT-derived additives improved all investigated characteristics, and only at the highest dosages (16% of ELT additives in total), excessive effects in the intermediate and low-temperature performance (<40 °C) were observed, resulting in strong decreases in phase angles. When the synthetic fiber was supplemented by the rubber powder, a significant improvement in the MSCR performance was seen under higher creep stresses, both in terms of non-recoverable compliance and percent recovery R%.

The asphalt mastics were also investigated for the stability of their properties under prolonged heating at 175 °C, relating to the production, transport, and paving processes of SMA mixtures. It was found that the evaluated properties improved most significantly during the first hour of heating and remained stable after the second hour of exposure to high temperature.

The observed effects of the ELT-derived additives could be linked to different interactions between the asphalt mastic (asphalt binder and mineral filler) and their fiber and rubber components. The synthetic fibers, with high melting points (>200 °C), can be believed to retain their structural integrity under the investigated conditions, but also some activity due to the applied surface treatments [50,51,52] should be expected. The purely mechanical effects due to the introduction of the fibrous component can possibly be explained by the following mechanisms:

- Introduction of additional solids (fibers), limiting the mobility of the binder;
- Binding of different areas of the asphalt mastic through individual and bundled fibers;
- Adsorption of asphalt binder on the surface of the fibers, reducing the amount of “free” binder;
- Individual fibers may introduce slippage planes under higher stresses.

As a result, increased internal friction and increased viscosity of the asphalt mastic was observed with the ELT-derived additives. The significant values of percent recovery R% seen in fiber-only formulations may be attributed to the substantial content of rubber particles in the fiber additive. Additionally, the surface treatments used in the fibers (most likely Resorcinol–Formaldehyde–Latex treatment, RFL) and diffusion of their natural/synthetic rubber components could potentially contribute to the improved elastic response observed in the investigated mastics. The rubber powder component of the ELT-derived additives also affects the properties of the asphalt mastics in different modes, mechanically and through interaction with light fractions of the bitumen:

- The rubber powder acts as an elastic aggregate, also limiting the mobility of the binder and adsorbing some of it;
- Rubber particles, particularly finer ones, may absorb some of the asphalt binder, resulting in swelling and digestion of maltene fractions from the asphalt binder, contributing to the increase in stiffness and elasticity of the asphalt mastic.

The experiments in this study were conducted on asphalt mastics, which due to the relative simplicity of the binder–filler–additive system offer advantages in terms of their testing. However, this simplification also introduces limitations regarding the direct applicability of the results to actual asphalt mixtures. In particular, the absence of coarse aggregates, air voids, and the associated structural interactions and volumetric relationships means that the more complex mechanical and rheological behavior of real mixtures cannot be captured. Consequently, the effects observed in this study may differ substantially from those seen in asphalt mixtures, depending on mixture type (e.g., asphalt concrete, stone mastic asphalt) and their volumetric composition.

Nevertheless, the findings from the conducted research provide some insights into the effects of ELT fibers and rubber powder on the mastic portion of the asphalt mixture. It can be firmly stated that the investigated ELT-derived materials significantly affected the properties of the asphalt mastics based on the paving-grade bitumen. These effects depended on the mode of loading, temperature, and loading time to which the investigated samples were subjected. Relatable results were obtained in a study by Calabi-Floody et al. [32] investigating asphalt mixtures with similar ELT-derived additives, where the greatest effects were seen at high and low testing temperatures—in resistance to permanent deformation and low-temperature cracking.

The presented results obtained through our work, as well as the studies conducted by other researchers, show that the use of these additives can be expanded beyond a simple remediation of binder drainage in SMA mixtures and, if developed further sufficiently, they could be utilized as components of functional additives, significantly improving the performance of asphalt mixtures.

## 5. Conclusions

Based on the experimental results and the analysis of the relevant literature, the following conclusions can be drawn:

- The incorporation of synthetic fibers and rubber powder derived from end-of-life tires (ELT) significantly modifies the rheological characteristics of asphalt mastics: improving high-temperature stiffness and elastic recovery while maintaining viable viscoelastic balance in many cases;
- The positive effects on the rheological properties suggest that the use of ELT-derived additives may result in increased resistance to permanent deformation and improved performance (and functional) characteristics;
- While increased additive content enhances elastic response, excessive dosing may reduce low-temperature flexibility and increase brittleness—the DSR testing has shown that a balanced formulation is required to ensure that both high- and intermediate-temperature performance are acceptable; this conclusion can be extrapolated with some caution to low-temperature performance;
- From a sustainability viewpoint, adoption of ELT-derived additives may reduce the need for polymer modifications in bituminous binders, improve durability of asphalt pavements, and support pavement material circularity.

Future work in this area will focus on the optimization of the properties of the additives, including modification of the rubber powder to improve the low-temperature performance and to evaluate its effects on the viscosity of the asphalt mastics at processing temperatures. Further investigations should aim to evaluate the effects on low temperature and cracking resistance, resistance to fatigue, and stiffness of asphalt mixtures. Additionally, investigations into the physical and chemical interactions between the components of the ELT-based additive should be carried out in an attempt to better understand and quantify the observed complex effects.

## Figures and Tables

**Table 1:** Characteristics of components from the processing of textile cord from ELTs.

Synthetic Fiber (F)				Rubber Powder (R)		
Material Composition	Average Fiber Length (mm)	Maximum Fiber Length (mm)	Rubber Powder Content (% Fiber Mass)	Source	Particle Size (mm)	Fiber Content (% of Rubber Powder Mass)
polyesters poliamides aramides viscose	2.9	13	24%	passenger car tires	0/0.8	15%

**Table 2:** Exemplary results of DSR testing representing the changes in the rheological properties of the selected asphalt mastics due to their prolonged exposure to high temperatures: norm of the complex shear modulus |G*|, phase angle δ, non-recoverable creep compliance J_nr_, and percent recovery R%.

Asphalt Mastic	Sample Heating Time (h)	|G*| (kPa)				δ (°)		J_nr_ (1/kPa)		R% (-)	
		20 °C	60 °C	80 °C	20 °C	60 °C	80 °C	3.2 kPa	25.6 kPa	3.2 kPa	25.6 kPa
1|F:0%|R:0%	0	11,771	20.2	1.78	61.8	83.5	87.9	0.211	480.31	0.039	0.005
	1	15,047	24.9	2.16	59.0	82.8	87.7	0.110	236.95	0.060	0.009
	2	15,119	26.3	2.27	58.9	82.8	87.5	0.129	297.93	0.053	0.007
3|F:0%|R:4%	0	14,644	40.7	3.86	56.0	78.4	84.9	0.074	0.136	0.253	0.060
	1	15,469	56.6	6.09	54.9	71.3	80.2	0.046	0.091	0.402	0.139
	2	18,064	53.2	5.92	54.7	71.0	79.2	0.040	0.089	0.475	0.178
6|F:8%|R:0%	0	14,104	70.2	12.40	52.6	68.0	60.7	0.023	0.066	0.606	0.244
	1	18,061	126.4	29.10	48.8	57.5	48.3	0.013	0.045	0.676	0.332
	2	16,167	119.9	27.03	53.3	59.0	51.4	0.008	0.022	0.759	0.486
8|F:8%|R:4%	0	16,836	134.2	33.16	49.4	58.3	47.2	0.007	0.019	0.755	0.455
	1	19,382	228.5	51.69	48.1	54.5	49.2	0.002	0.004	0.856	0.734
	2	19,188	252.4	56.39	47.7	53.7	49.4	0.001	0.003	0.884	0.790

**Table 3:** Fitting parameters of dynamic shear modulus mastercurves of asphalt mastics with different amounts of ELT-derived additives.

Asphalt Mastic	ν	α	β	γ	C_1_	C_2_	Lower Modulus Asymptote (kPa)	Upper Modulus Asymptote (kPa)	R^2^
1|F:0%|R:0%	−4.600	10.583	−0.456	−0.360	9.94	132.74	2.51 × 10^−5^	9.61 × 10^5^	0.9989
2|F:4%|R:0%	−2.505	8.344	−0.171	−0.388	10.03	132.50	3.12 × 10^−3^	6.90 × 10^5^	0.9986
3|F:0%|R:4%	−5.881	12.046	−0.693	−0.288	9.75	127.21	1.31 × 10^−6^	1.46 × 10^6^	0.9993
4|F:5%|R:2.5%	−1.064	6.885	−0.014	−0.371	11.33	143.07	8.64 × 10^−2^	6.63 × 10^5^	0.9992
5|F:0%|R:8%	−4.777	10.826	−0.703	−0.277	10.35	128.31	1.67 × 10^−5^	1.12 × 10^6^	0.9972
6|F:8%|R:0%	0.608	4.788	0.531	−0.496	14.77	172.47	4.06 × 10^0^	2.49 × 10^5^	0.9962
7|F:4%|R:8%	−1.563	7.379	−0.271	−0.309	10.59	129.29	2.73 × 10^−2^	6.53 × 10^5^	0.9852
8|F:8%|R:4%	0.151	5.472	0.167	−0.382	11.65	140.60	1.42 × 10^0^	4.20 × 10^5^	0.9991
9|F:8%|R:8%	0.227	5.450	0.088	−0.355	13.75	152.22	1.68 × 10^0^	4.75 × 10^5^	0.9992

**Table 4:** Fitted parameters of statistical models for predicting dynamic shear modulus of asphalt mastics with ELT-derived additives (1.59 Hz).

	Dependent Variable: log_10_(|G*|)								
Temperature	10 °C	20 °C	30 °C	40 °C	50 °C	60 °C	70 °C	80 °C	90 °C
Intercept	4.771 ***	4.160 ***	3.444 ***	2.720 ***	1.953 ***	1.313 ***	0.743 ***	0.26 **	−0.148
F	−0.208	0.979	2.597 **	4.364 ***	7.808 ***	10.754 ***	14.435 ***	18.016 ***	21.834 ***
R	0.134	1.277	2.917 ***	4.658 ***	11.794 ***	14.064 ***	15.285 ***	15.582 ***	15.413 ***
F^2^	-	-	-	-	−8.175	−15.492	−28.569	−41.014	−56.902
R^2^	-	-	-	-	−50.824 *	−54.127 **	−48.827 *	−42.475	−37.881
F:R	7.802	2.816	−1.032	−6.222	−37.898 **	−56.163 ***	−73.214 ***	−84.513 ***	−92.866 **
Adj. R^2^	0.143	0.528	0.824	0.901	0.952	0.968	0.969	0.956	0.935

Statistical significance: ‘***’ *p* < 0.001; ‘**’*p* < 0.01; ‘*’*p* < 0.05.

**Table 5:** Fitted parameters of statistical models for predicting phase angle of asphalt mastics with ELT-derived additives (1.59 Hz).

	Dependent Variable: δ								
Temperature	10 °C	20 °C	30 °C	40 °C	50 °C	60 °C	70 °C	80 °C	90 °C
Intercept	51.438 ***	59.005 ***	65.934 ***	71.757 ***	78.013 ***	82.943 ***	84.424 ***	84.889 ***	87.477 ***
F	−88.614 **	−102.962 **	−121.065 **	−142.53 *	−278.16 ***	−399.782 ***	−388.364 ***	−473.11 ***	−942.511 ***
R	−71.89 **	−88.407 *	−130.477 **	−185.947 **	−287.503 ***	−291.177 **	−250.032 ***	−197.04 *	−78.488
F^2^	−270.556	−262.453	−244.489	−335.747	619.411	1130.223	-	-	4717.963 *
R^2^	−237.511	−209.27	17.296	214.896	711.319	231.33	-	-	−1030.968
F:R	825.202 ***	767.903 **	784.963 *	1017.535 *	1697.747 ***	2200.029 **	2326.76 *	2356.40	2423.363
Adj. R^2^	0.936	0.932	0.929	0.918	0.949	0.915	0.833	0.763	0.785

Statistical significance: ‘***’ *p* < 0.001; ‘**’*p* < 0.01; ‘*’*p* < 0.05.

**Table 6:** Fitted parameters of statistical models for predicting non-recoverable compliance (log10(Jnr)) of asphalt mastics with ELT-derived additives (at 60 °C).

	Dependent Variable: log_10_(J_nr_)					
Creep Stress (kPa)	0.1	1.6	3.2	6.4	12.8	25.6
Intercept	−0.920 ***	−0.886 ***	−0.853 ***	−0.783 ***	−0.679 ***	−0.518 ***
F	−20.729 ***	−20.553 ***	−19.309 ***	−17.759 ***	−15.645 ***	−14.131 ***
R	−15.301 ***	−15.120 ***	−16.180 ***	−17.046 ***	−17.654 ***	−18.859 ***
F^2^	94.688 *	93.156 **	80.516 *	65.846	48.076	41.061
R^2^	27.777	25.759	35.287	38.558	44.601	57.797
F:R	−24.389	−24.731	−22.398	−26.842	−37.441	−50.520 *
Adj. R^2^	0.958	0.958	0.961	0.961	0.963	0.971

Statistical significance: ‘***’ *p* < 0.001; ‘**’ *p* < 0.01; ‘*’ *p* < 0.05.

**Table 7:** Fitted parameters of statistical models for predicting percent recovery (R%) of asphalt mastics with ELT-derived additives (at 60 °C).

	Dependent Variable: R%					
Creep Stress (kPa)	0.1	1.6	3.2	6.4	12.8	25.6
Intercept	0.156 *	0.117 ***	0.079 ***	0.030	−0.027	−0.059.
F	17.268 ***	15.175 ***	14.694 ***	13.620 ***	11.609 ***	8.438 ***
R	11.349 **	9.403 ***	9.702 ***	9.895 ***	9.319 ***	7.662 ***
F^2^	−122.026 **	−95.276 ***	−85.996 ***	−73.895 ***	−56.578 ***	−40.107 *
R^2^	−62.166	−36.582 **	−33.944 ***	−31.789 ***	−25.436 *	−17.272
F:R	−54.367 *	−56.752 ***	−59.510 ***	−52.774 ***	−33.292 ***	6.147
Adj. R^2^	0.749	0.968	0.982	0.987	0.971	0.956

Statistical significance: ‘***’ *p* < 0.001; ‘**’ *p* < 0.01; ‘*’ *p* < 0.05.

## Data Availability

The original contributions presented in this study are included in the article. Further inquiries can be directed to the corresponding author.
